# The Relationship between Maternal Atopy and Childhood Asthma in Pretoria, South Africa

**DOI:** 10.1155/2013/164063

**Published:** 2013-01-27

**Authors:** Salome Abbott, Piet Becker, Robin J. Green

**Affiliations:** ^1^Department of Paediatrics and Child Health, University of Pretoria, Steve Biko Academic Hospital, Pretoria, South Africa; ^2^Department of Biostatistics, Medical Research Council of South Africa and Department of Paediatrics and Child Health, University of Pretoria, Pretoria, South Africa

## Abstract

*Introduction*. Asthma is the commonest chronic condition of children. Diagnosis of this condition remains difficult. Many surrogate markers are used, such as documenting evidence of atopy. *Method*. A random sample of asthmatic children and their mothers attending the Children's Chest and Allergy Clinic at Steve Biko Academic Hospital were enrolled. Children were classified as having atopic or nonatopic asthma. Mothers completed a questionnaire to uncover atopic features. *Results*. Along with their mothers, 64 children with atopic asthma and 36 with nonatopic asthma were studied. The proportion of children with atopic asthma does not differ for mothers with and without a positive SPT (*P* = 0.836), a history of asthma (*P* = 0.045), symptoms suggestive of an allergic disease (*P* = 1.000), or who were considered to be allergic (*P* = 0.806). The odds ratio of a child having atopic asthma when having a mother with a doctor diagnosed history of asthma is 4.76, but the sensitivity is low (21.9%). *Conclusion*. The data demonstrates that all maternal allergic or asthmatic associations are poor predictors of childhood atopic asthma. Despite the increased risk of atopic asthma in a child to a mother that has a doctor diagnosis of asthma (OR 4.76 *P* = 0.045), this is a poor predictor of atopic asthma (sensitivity 21.9%).

## 1. Introduction

Asthma is one of the commonest childhood illnesses. Unfortunately, in some individuals, the diagnosis remains difficult, particularly in the preschool wheezer. This leads to widespread underdiagnosis, which negatively affects the quality of life of asthmatic children.

In an attempt to provide insight into the wheezy infant, much research has been conducted in order to provide markers, that may help predict and aid in the diagnosis of asthma in young individuals. Despite this, the epidemiology and disease expression of asthma and other allergic diseases still remain poorly understood. In the Northern Hemisphere, the relationship between asthma and atopy has been clearly shown [1, 2]. For this reason, the presence of atopy in children is often used as a surrogate marker to assist in making the diagnosis of asthma [2].

In the developing world and particularly in the South African context, the relationship between atopy and asthma may not be as clear [3]. Since 2005 the atopic status of asthmatic children attending the Steve Biko Academic Hospital Paediatric Asthma Clinic has been investigated. Results have demonstrated that only 49% of the children with asthma had one or more positive skin prick tests to common aero-allergens [4]. This is much lower than the atopic rate of asthmatics reported in the Northern Hemisphere [5]. This suggests that these children had a lower rate of an atopic diathesis than other groups reported, suggesting that using atopy as a diagnostic tool in our context may not be a perfect marker for asthma.

While the genetic basis for asthma remains unclear, the interaction between genetics and the environment is even more illusive [6]. The role of the environment has been postulated to explain the increase in the prevalence of asthma [7–11], as well as other allergic diseases. Some of these environmental factors include urbanisation, dietary changes, vitamin and micronutrient insufficiency, changes in microbial burden, and industrial pollution. It is possible that these environmental factors play a greater role in the aetiology and expression of asthma and other allergic diseases in our context.

This study was performed to further investigate the relationship between maternal atopy and asthma. It has important implications because identification of atopy in relatives of asthmatic children may be unhelpful in the diagnosis of childhood asthma, especially in our setting, since fewer asthmatic patients are atopic. It is important to provide practitioners with diagnostic tools to aid in making a diagnosis, so as not to waste time asking questions that do not provide information.

In the context of this study, the following definitions are used.

 
*Family history of allergy*: family member with symptoms suggestive of an allergic disease or a known allergic disease, for example, allergic rhinitis, asthma, and food allergy.
 
*Atopy*: positive skin prick test to common aero-allergens and/or foods, in the context of an allergic disease [12].
 
*Atopic asthmatic*: asthmatic with a positive skin prick test.
 
*Nonatopic asthmatic*: asthmatic with a negative skin prick test.
 
*“Allergic” mom*: presence of a positive skin prick test, or a history of allergic symptoms or disease.

## 2. Objective

To determine if a maternal history of allergic disease or symptoms of such a disease and/or atopy is a useful predictor of the allergic basis of her child's asthma. This was performed by determining

- the association of atopy in mothers with atopic asthma in their children,
- the association of asthma in mothers with atopic asthma in their children, and
- the association of maternal history of allergic symptoms in mothers with atopic asthma in their children.

## 3. Methods

A random sample of children and their mothers attending the Children's Chest and Allergy Clinic at Steve Biko Academic Hospital, in Pretoria, South Africa, were enrolled. Children diagnosed with asthma and known to the clinic for longer than six months were considered. A diagnosis of asthma was made by all of the following [13]:

1. a history of respiratory symptoms which respond to bronchodilators,
2. a history of respiratory symptoms which respond to inhaled or oral corticosteroids, and
3. airway hyper responsiveness, as demonstrated by reversible spirometry.

The study was approved by the Research Ethics Committee of the Faculty of Health Sciences of the University of Pretoria. Maternal consent and patient assent was obtained. Skin-prick testing (SPT) or ImmunoCAP (Thermofisher) test results, to common aeroallergens and foods, of the children were obtained from the child's hospital records.

Mothers completed a detailed questionnaire which included demographic details, birth history, occupation, habits, present environment, medical history, symptomatology of atopy, and a history of symptoms suggestive of allergic diseases. These were subdivided into skin, upper and lower respiratory tract symptoms, such as allergic rhinitis, sinusitis, eczema and asthma, as well as any history of proven asthma.

SPT using the Alk-Abelló allergen extracts (Laboratory Specialities) and negative (0.5% phenol) and positive (1% histamine) controls were performed on the mothers. The aero-allergen extracts used were Bermuda grass, corn pollen, 5 grass mix, tree mix, *Candida albicans*, *Aspergillus fumigatus*, cat-hair epithelium, dog-hair dander, feather mix, house-dust mix, and standardised *Dermatophagoides pteronyssinus*. Each extract was applied to the volar surface of the forearm with a sterile prick lancette. Reactions were read at 10 minutes, any wheal 3 mm or greater than the negative control was regarded as positive.

### 3.1. Statistical Analysis

The results were captured and the proportions were compared using the Fisher's-exact test. Test characteristics were evaluated for sensitivity, specificity, and predictive values using a standard two by two table. The outcome of interest was asthmatic children with a positive SPT (used to distinguish between atopic and nonatopic asthma).

## 4. Results

100 children and their parents were enrolled. All children were under 12 years old. The age range was 1–12 years (mean 6 years). Along with their mothers, 64 children with atopic asthma and 36 with nonatopic asthma were studied. The patients were not stratified according to race. The proportion of children with atopic asthma does not differ significantly for mothers with and without a positive SPT (*P* = 0.836; 0.625 (30/48) versus 0.654 (34/52)). The proportion of children with atopic asthma does not differ significantly for mothers with and without a history of doctor diagnosed asthma (*P* = 0.045; 0.875 (14/16) versus 0.595 (50/84)). The proportion of children with atopic asthma does not differ significantly for mothers with and without symptoms suggestive of an allergic disease (*P* = 1.000; 0.643 (45/70) versus 0.633 (19/30)). The proportion of children with atopic asthma does not differ significantly for mothers who were considered to be allergic (presence of a positive skin prick test, or a history of allergic symptoms) or not (*P* = 0.806; 0.649 (50/77) versus 0.608 (14/23)). The diagnostic variables (sensitivity, specificity, and predictive values) are reflected in Table 1.

The relative risk (odds ratio) of a child having atopic asthma when having a mother with or without the exposure variable is reflected in Table 2.

## 5. Discussion

The data in Table 1 demonstrates that all maternal allergic or asthmatic associations are poor predictors of atopic asthma in their children. The descriptive statistics is low for all maternal factors. Only the association of maternal doctor diagnosed asthma reached statistical significance (*P* = 0.045) but this may have limited clinical significance.

Despite the increased risk of atopic asthma in a child of a mother that has a doctor diagnosis of asthma (OR 4.76 *P* = 0.045), a mother with a doctor diagnosis of asthma is, nevertheless, a poor predictor of a child with atopic asthma (sensitivity 21.9%). The false negative rate of this predictive test is high.

The findings in this study are important because the literature suggests that the most reliable way to demonstrate the inherited tendency of asthma is to demonstrate a positive family history of atopy. This is why atopy is used as a surrogate marker to aid in the diagnosis of childhood asthma. But it is clear here, that a history of maternal atopy or allergic diseases is not a good predictor of childhood asthma in our cohort of patients.

It has previously been suggested that the reason for the poor association between family history of allergic disease and childhood asthma may be due to lack of diagnosis of allergic diseases in family members [14]. This is because of underreporting of these symptoms by patients, or failure of doctors to recognise and diagnose allergic diseases. Parents should rather be asked about specific symptoms suggestive of asthma, allergic rhinitis, and other allergic diseases [14]. However, this study demonstrates that this cannot be the whole explanation for a poor family history, since mothers were specifically questioned on symptomatology suggestive of allergic diseases. Therefore adequate history taking is not a simple solution to a complex problem.

Perhaps the relationship between asthma and atopy has been overestimated in the first world [15]. Or perhaps this relationship, which we rely upon in everyday practice to aid in the diagnosis of childhood asthma, cannot be extrapolated to the third world. Other risk factors for asthma in our setting may have been neglected.

## 6. Conclusion

Atopic disease expression carries an inherited or genetic component [16]. Despite this, in our study, maternal atopy (positive SPT and an allergic disease process), or a history of symptoms suggestive of allergic diseases were not good predictors of atopic asthma in children. Even previously doctor diagnosed maternal asthma may not be a useful predictor of atopic asthma in her child. This supports previous local studies that have demonstrated that atopy occurs in fewer asthmatic children, at best in South Africa, than previously thought.

This raises questions about the uniform association between allergy and asthma, especially in Africa and suggests that asthma may be associated with other aetiological factors. Some environmental factors postulated are urbanisation, dietary changes, vitamin and micronutrient insufficiency, changes in microbial burden, and industrial pollution.

The data obtained are the result of a pilot study based on a limited number of patients. Also, reliance on maternal history to differentiate allergic from nonallergic mothers is another important limitation. These results should therefore be confirmed by further studies. Families should also be stratified according to race, to determine whether the results are influenced. The information obtained however, together with other local studies, has shown the emergence of a new picture of childhood asthma in the third world context. This is different to what was previously thought. Currently, the diagnosis of asthma in young children is aided by a family history of atopy or allergic diseases. But this may not be appropriate in the South African setting. There are many unanswered questions. This provides the motivation for further studies to be conducted among different ethnic groups, especially in South Africa. This will hopefully shed more light on the complexity of the epidemiology and aetiology of childhood asthma in the third world.

## Figures and Tables

**Table 1:** Descriptive statistics for the relationship between maternal allergy or asthma and children with atopic asthma.

Prevalence of maternal	Doctor diagnosed asthma	Skin prick test positive	Allergic symptoms	Allergic symptoms or SPT positive
Sensitivity	21.9%	46.9%	70.3%	78.1%
Specificity	94.4%	50%	30.7%	25%
ROC area	0.582	0.484	0.504	0.516
Positive predictive value (PPV)	87.5%	62.5%	64.3%	64.9%
Negative predictive value (NPV)	40.5%	34.6%	36.7%	39.1%

**Table 2:** Odds ratio of a child having atopic asthma when having a mother with or without the exposure variable.

	Skin prick test positive	Doctor diagnosed asthma	Allergic symptoms	Allergic symptoms or SPT positive
Odds ratio (OR)	0.882	4.76	1.04	1.19
95% CI	(0.36; 2.16)	(0.98; 45.3)	(0.38; 2.75)	(0.39; 3.42)
